# In the field: exploiting the untapped potential of immunogenic modulation by radiation in combination with immunotherapy for the treatment of cancer

**DOI:** 10.3389/fonc.2012.00104

**Published:** 2012-09-06

**Authors:** Anna R. Kwilas, Renee N. Donahue, Michael B. Bernstein, James W. Hodge

**Affiliations:** ^1^Laboratory of Tumor Immunology and Biology, Center for Cancer Research, National Cancer Institute, National Institutes of HealthBethesda, MD, USA; ^2^Department of Radiation Oncology, Albert Einstein College of MedicineNew York, NY, USA

**Keywords:** radiation therapy, cancer immunotherapy, cancer vaccine, abscopal effect

## Abstract

Radiation has long been the standard of care for many types of cancer. It is employed to locally eradicate tumor cells as well as alter tumor stroma with either curative or palliative intent. Radiation-induced cell damage is an immunologically active process in which danger signals are released that stimulate immune cells to phagocytose and present locally released tumor-associated antigens (TAAs). Recent studies have indicated that radiotherapy can also alter the phenotype of cancer cells that remain after treatment. These cells upregulate TAAs as well as markers, including major histocompatibility complex and costimulatory molecules, that make them much more immunostimulatory. As our understanding of the immunomodulatory effects of radiation has improved, interest in combining this type of therapy with immune-based therapies for the treatment of cancer has grown. Therapeutic cancer vaccines have been shown to initiate the dynamic process of host immune system activation, culminating in the recognition of host cancer cells as foreign. The environment created after radiotherapy can be exploited by active therapeutic cancer vaccines in order to achieve further, more robust immune system activation. This review highlights preclinical studies that have examined the alteration of the tumor microenvironment with regard to immunostimulatory molecules following different types of radiotherapy, including external beam radiation, radiolabeled monoclonal antibodies, bone-seeking radionuclides, and brachytherapy. We also emphasize how combination therapy with a cancer vaccine can exploit these changes to achieve improved therapeutic benefit. Lastly, we describe how these laboratory findings are translating into clinical benefit for patients undergoing combined radiotherapy and cancer vaccination.

## RATIONALE FOR COMBINING RADIATION AND IMMUNOTHERAPY

Radiation therapy (RT) is an integral component of cancer care. A recent article in the *Journal of Clinical Oncology* reported that the demand for RT during the initial course of cancer treatment is expected to increase by 22% (from 470,000 patients receiving RT in 2010 to 575,000 in 2020) as a result of the aging and diversification of the U.S. population (Smith et al., 2010). Depending on the presentation and site of disease, RT can have either a curative or palliative intent. In the traditional view, ionizing radiation causes cancer cell death through irreparable DNA damage, which results in apoptosis or failure to progress through the cell cycle. An additional consequence of RT that has sparked significant interest is its effects on cells not killed by RT and the resulting impact on the immune system. Here, we review the immunogenic nature of radiation in preclinical models as well as in the clinic. We also provide a rationale for combining RT with immunotherapeutic approaches.

Several studies have shown the various mechanisms by which RT stimulates the immune system. One vital by-product of radiation damage to tumors is the exposure of a large amount of tumor antigens, in the form of necrotic and apoptotic tumor cells and cellular debris, to the immune system (Melcher et al., 1999; Chen et al., 2001; Kotera et al., 2001). The increased availability of released tumor-associated antigens (TAAs) for uptake by circulating dendritic cells (DCs) and other antigen-presenting cells (APCs) can result in tumor-specific immune attack. One report confirmed that irradiating tumors expressing low levels of antigen caused sufficient release of antigen to sensitize tumor stromal cells to destruction by cytotoxic T lymphocytes (CTLs; Zhang et al., 2007). In addition to causing the release of TAAs, RT also creates an inflammatory milieu by inducing the expression of several proinflammatory cytokines, including IL-1β and TNF-α (Hallahan et al., 1989; Ishihara et al., 1993; Hong et al., 1999; Demaria et al., 2005). Increased expression of these cytokines has been linked to tumor regression, growth inhibition, and tumor-cell death. Furthermore, upregulation of major histocompatibility complex (MHC) molecules, costimulatory molecules, adhesion molecules, and death receptors in tumor cells, surrounding stroma, and vascular endothelium following irradiation can also potentiate CD8^+^ cytolytic responses (Friedman, 2002; McBride et al., 2004; Demaria et al., 2005; Nesslinger et al., 2007). Similarly, radiation-induced damage can upregulate expression of vascular cell adhesion molecule 1 (VCAM-1) on tumor vessels, thus facilitating T cell migration (Lugade et al., 2005). Cytokine release by irradiated tumor cells can also increase T cell infiltration into the tumor microenvironment (Matsumura et al., 2008). Other reports have focused on the release of “danger” signals in response to ionizing radiation, which may link initial non-specific immune responses to the development of specific adaptive immunity (McBride et al., 2004). Two such signals that can promote antitumor immune responses after irradiation include the translocation of calreticulin to the cell surface (Obeid et al., 2007) and the release of high-mobility group box 1 (HMGB1) by dying tumor cells, which can activate DCs through Toll-like receptor 4 (Apetoh et al., 2007).

Although the most common form of RT, external beam radiation therapy (EBRT), is conventionally administered in fractionated doses, it is unclear what the optimal dose schedule for EBRT should be when it is combined with immunotherapy. Recent studies have focused on the importance of the dose and fractionation of EBRT in modulating the immune system in order to answer this question. As opposed to conventional RT, stereotactic body radiotherapy takes advantage of technological advances to allow for highly precise administration of ablative doses of RT to tumors, while avoiding damage to the surrounding organs. Lee et al. (2009) used a murine model to show that doses of RT (15–25 Gy ×1 fraction) alone generated robust CD8^+^ T cell-dependent immunity that led to tumor reduction, reduced relapse of primary tumor, and eradication of metastasis in some settings. This group concluded that the fractionation and dose schedule examined successfully disrupted physical and immunologic barriers, introduced danger signals, increased DC cross-presentation of tumor antigen, and possibly reversed T cell unresponsiveness in tumor-bearing hosts, leading to the rejection of local and distal tumors. In a similar study, mice bearing OVA-expressing B16-F0 tumors that were treated with a total dose of 15 Gy of localized RT delivered in a single fraction had enhanced APC trafficking to draining lymph nodes and greater capability to present tumor antigens compared to non-irradiated mice. This led to increased numbers of tumor-specific T cells that secreted IFN-γ upon peptide stimulation within tumor-draining lymph nodes and improved lysis of tumor-cell targets (Lugade et al., 2005). A report by Schaue et al. (2011) not only reinforced the importance of dose and fractionation, but also highlighted the delicate balance between the immunostimulatory and immunosuppressive effects of radiation. In this study, mice bearing B16-OVA murine melanoma were treated with up to 15 Gy of radiation, given in various size fractions. Subsequent observation of tumor growth revealed that after single doses, tumor control increased with the size of radiation dose, as did the number of tumor-reactive T cells. However, this was offset at the highest dose by an increase in regulatory T cells (Tregs), which are known to suppress tumor-specific immunity (Nishikawa and Sakaguchi, 2010). Fractionated treatment with medium-size radiation doses of 7.5 Gy/fraction resulted in the best tumor control and tumor immunity, while maintaining low Treg numbers (Schaue et al., 2011). Taken together, these results indicated that greater doses of RT delivered in fewer fractions can generate tumor-specific immune responses similar to that of lower doses given more frequently, although a threshold level above which the balance shifts toward immunosuppression may exist. Interestingly, preclinical studies suggest that modalities of RT other than EBRT are able to modulate tumor phenotype and enhance T cell-mediated killing. These modalities include bone-seeking radionuclides, radiolabeled monoclonal antibodies (mAbs), and brachytherapy, all of which will be discussed later in this review.

In addition to the preclinical data presented above, there is substantial clinical evidence of radiation-induced immune activation. Nesslinger et al. (2007) evaluated pre- and post-treatment serum samples from 73 men with non-metastatic prostate cancer and described the development of treatment-associated autoantibody responses in nearly 14% of patients treated with EBRT and 25% who received brachytherapy, compared with 0 of 14 patients who underwent radical prostatectomy. In agreement with their preclinical findings, Schaue et al. (2008) observed that tumor-specific T cells clearly increase in most colorectal cancer patients after completion of chemoradiation therapy and in most prostate cancer patients after RT. Of note, levels of Tregs increased in colorectal cancer patients following treatment, again suggesting a potential threshold above which immunosuppressive effects may dominate. In a recent case report published in *The New England Journal of Medicine*, a patient suffering from metastatic melanoma with disease progression on ipilimumab (IPI, Yervoy; Bristol-Myers Squibb), a mAb that inhibits CTL-associated antigen 4 (CTLA-4), an immunologic checkpoint on T cells, showed a favorable response only after receiving local RT for a metastatic spinal lesion (Postow et al., 2012). The patient experienced out-of-field tumor shrinkage, with antibody responses to tumor-specific antigens, changes in peripheral-blood immune cells, and increases in antibody responses to other antigens. These findings highlight a rare but important phenomenon known as the abscopal effect, where local RT elicits a systemic response and causes tumor regression at a site distant from the irradiated field. The abscopal effect has also been reported in tumors other than melanoma, such as lymphomas, hepatocellular carcinoma, and certain adenocarcinomas (Ehlers and Fridman, 1973; Antoniades et al., 1977; Rees and Ross, 1983; Ohba et al., 1998).

**FIGURE 1:**
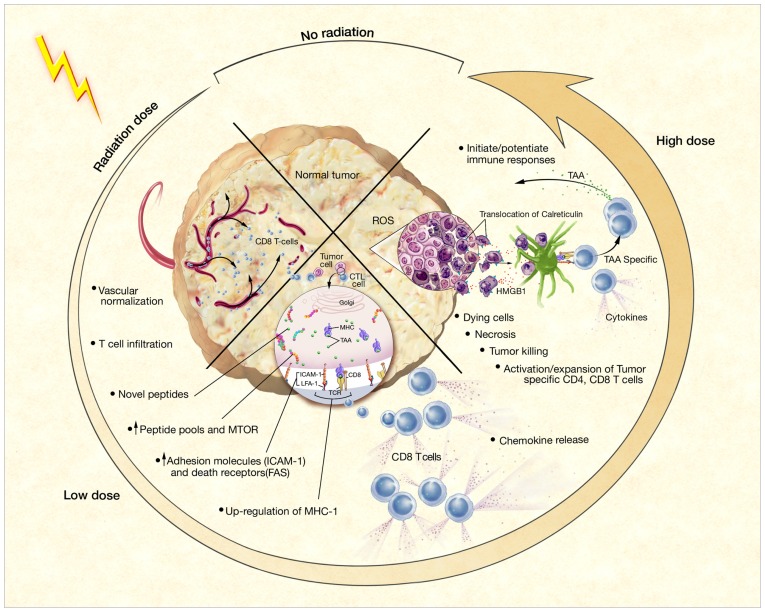
**Phenotypic and microenvironmental changes in tumors elicited by RT that can be exploited by immunotherapy (Hodge et al., 2008)** Each of the potential mechanisms of RT-induced immunogenic modulation shown here is discussed further and referenced in the text.

Taken together, these data indicate that RT effectively stimulates immune responses by increasing the production of inflammatory cytokines, causing the release of large amounts of tumor antigen, enhancing antigen processing and presentation, improving T cell migration to sites of disease, and activating tumor-specific CTLs (**Figure 1**). As described above, this activation may translate into both local and systemic clinical benefit. Nevertheless, a large tumor burden often creates enough immune suppression to prevent successful immune intervention. In this case, studies have proposed that local RT can also sufficiently reduce tumor burden to allow for further therapeutic intervention by immunotherapy, such as vaccination or blockade of inhibitory molecules, and, in some cases, may synergize with such therapy (Kamrava et al., 2009). By enhancing the frequency, magnitude, and character of the immune responses induced by RT with immunomodulatory agents, cancer patients could experience further improved outcomes.

## PRECLINICAL EVIDENCE OF SYNERGY WHEN RADIATION AND IMMUNOTHERAPY ARE COMBINED

Several recent studies have indicated that radiation-induced cell death is an immunologically active process. This is demonstrated in one way because radiation-induced cell death causes the release of TAAs that can potentially be exploited to stimulate robust tumor-specific immune responses (Hannani et al., 2011). On their own, tumor cells typically do not generate potent antitumor immune responses due to their inefficient expression of molecules that are critical for antigen processing and presentation, such as the antigen transporter gene product TAP-2, MHC class I molecules, and T cell costimulatory molecules such as B7-1 (CD80; Sanda et al., 1995). However, radiation-induced cell death results in the release of novel TAAs that can be taken up, processed, and presented by APCs in the tumor microenvironment and draining lymph nodes. Reits et al. (2006) demonstrated that RT increases the peptide repertoire available for MHC class I molecules to present to CTLs, not only by increasing the degradation of existing proteins, but by activating the mammalian target of rapamycin pathway, leading to increased protein translation and creation of a novel peptide repertoire. Irradiation has additionally been shown to induce the expression of membrane-bound calreticulin on tumor cells, which acts as a recognition and phagocytosis signal for DCs. It can also induce the release of “danger signals” for DC activation, such as various heat shock proteins and HMGB1 (Demaria et al., 2005; Tesniere et al., 2008). Friedman (2002) has previously described a “danger model” of immunity, wherein ionizing radiation generates an inflammatory microenvironment filled with apoptotic and necrotic cells, chemokines, cytokines, and other inflammatory mediators. This inflammatory milieu is believed to activate APCs and support their processing of newly exposed TAAs.

Although RT is traditionally employed to destroy tumor cells, some of the cells within a given tumor mass receive doses of radiation that do not result in cell death because of the need to limit damage to normal tissues. A number of preclinical studies have shown that these lower doses of radiation are capable of inducing phenotypic changes within tumor cells that ultimately facilitate immune-cell recognition and immune-mediated tumor killing. Molecules reported to be altered by such doses of radiation include TAAs, MHC class I, Fas/CD95, and the costimulatory molecules B7-1, lymphocyte function-associated antigen 3 (LFA-3), and intercellular adhesion molecule 1 (ICAM-1; Vereecque et al., 2000; Vondracek et al., 2001; Chakraborty et al., 2003; Garnett et al., 2004; Reits et al., 2006; Ifeadi and Garnett-Benson, 2012). These molecules are well known to play a role in CTL-mediated killing. MHC class I is responsible for direct presentation of tumor antigen peptides to CTLs, while increased numbers of adhesion molecules improve cell-to-cell attachment, enhancing the ability of a T cell to kill its target (Zamai et al., 1994; Baluna et al., 2006; Reits et al., 2006). Fas-mediated apoptosis plays an important role in CTL-mediated tumor-cell destruction, with interaction of the Fas ligand on activated CTLs with the Fas receptor on the target cell, inducing apoptosis of the target cell.

Using a murine adenocarcinoma cell line transfected to express carcinoembryonic antigen (CEA, MC38-CEA), Chakraborty et al. (2003) demonstrated *in vitro* that irradiation enhanced the surface expression of two molecules involved in T cell-mediated immune attack, Fas/CD95 and ICAM-1, in a dose-dependent manner. Moreover, they reported that exposure to radiation (20 Gy) enhanced the sensitivity of this murine cell line to antigen-specific CTL killing by up to fourfold, and that this increase in CTL sensitivity was shown to be via the Fas/Fas ligand pathway (Chakraborty et al., 2003). A follow-up study examined whether this phenomenon similarly occurs in human cancer cells. Utilizing a variety of human carcinoma cell lines (12 colon, 7 lung, and 4 prostate), Garnett et al. (2004) investigated whether 10 or 20 Gy of gamma radiation could alter the cell surface expression of a variety of molecules involved in T cell-mediated immune attack, including Fas/CD95, adhesion molecules, MHC class I, and TAAs such as CEA and mucin-1 (MUC-1). They found that at least one of these molecules was upregulated in 91% of the cell lines post-irradiation (Garnett et al., 2004). Moreover, five of five irradiated CEA^+^, HLA-A2^+^ colon cancer cell lines demonstrated significantly enhanced killing by CEA-specific HLA-A2-restricted CD8^+^ CTLs compared to non-irradiated controls (Garnett et al., 2004). Modrak et al. (2003) also showed an increase in TAA expression among irradiated colon cancer cell lines. These *in vitro* studies collectively demonstrated that RT can make both mouse and human tumor cells more amenable to immune recognition and attack.

Another clinically relevant form of radiation, bone-seeking chelated radionuclide, is similarly capable of inducing phenotypic changes within tumor cells, thereby enabling immune-cell recognition and enhancing CTL killing. Chakraborty et al. (2008b) evaluated the FDA-approved bone-seeking radionuclide samarium-153 (^153^Sm-EDTMP; Quadramet^®^, Cytogen), used as palliation for pain caused by metastatic bone lesions, for its ability to change the phenotype of tumor cells. The calculated dose of radiation delivered to bone metastases by this agent is between 18 and 80 Gy (Eary et al., 1993; Maini et al., 2004). In this study, 10 human tumor cell lines representing classes of tumors that metastasize to bone (four prostate, two breast, four lung) were exposed to clinically relevant levels of ^153^Sm-EDTMP for 4 days, then examined by flow cytometry for modulation of several cell surface molecules. Of the 10 cell lines, 100% upregulated Fas and CEA, 70% upregulated MUC-1, 40% upregulated MHC class I, and 30% upregulated ICAM-1. Exposure of the prostate cancer cell line LNCaP to ^153^Sm-EDTMP also resulted in upregulation of prostate-specific antigen (PSA), prostate-specific membrane antigen (PSMA), and prostatic acid phosphatase (PAP). Additionally, treatment of LNCaP cells with ^153^Sm-EDTMP rendered them more susceptible to killing by a variety of antigen-specific CTLs. These preclinical data suggest that ^153^Sm-EDTMP may work synergistically with immunotherapy to increase the susceptibility of tumor cells to CTL killing, and have formed the basis for an ongoing clinical trial.

These and other preclinical studies have collectively demonstrated that radiation can be utilized to make tumor cells more amenable to immune recognition and attack, and form the rational basis for the combinatorial use of local tumor irradiation and immunotherapy. A number of preclinical studies have demonstrated that localized treatment of tumors with lower doses of EBRT acts synergistically with immunotherapy to enhance antitumor immune responses. Chakraborty et al. (2003) demonstrated that EBRT (8 Gy) of subcutaneous MC38-CEA tumors markedly enhanced the efficacy of immunotherapy in the form of CTL adoptive transfer. In this study, C57B6 mice were implanted subcutaneously on the hind leg with MC38-CEA cells. Nine days later, mice were randomized to receive no treatment, EBRT of the tumor alone, adoptive transfer of CEA-specific CTLs alone, or the combination of both EBRT and adoptive transfer. EBRT alone and adoptive transfer alone ultimately failed to significantly impact tumor growth in these mice relative to untreated controls (Chakraborty et al., 2003). However, treatment of tumors with the combination of EBRT and CTL adoptive transfer resulted in a significant reduction in tumor growth rate and volume relative to mice receiving either no treatment or EBRT or CTL adoptive transfer alone. Moreover, 50% of mice receiving the combination treatment remained tumor-free for the duration of the experiment (40 days; Chakraborty et al., 2003). In a similar study by Reits et al. (2006), mice were implanted with MC38 tumor cells. When tumors became established, mice received EBRT (10 Gy) and/or adoptive transfer of gp70-specific CTLs (Reits et al., 2006). Neither radiation nor adoptive transfer alone was curative; however, the combination of local irradiation of the tumor and adoptive transfer of CTLs significantly reduced tumor burden and, in most mice, completely eradicated the tumor mass.

**FIGURE 2:**
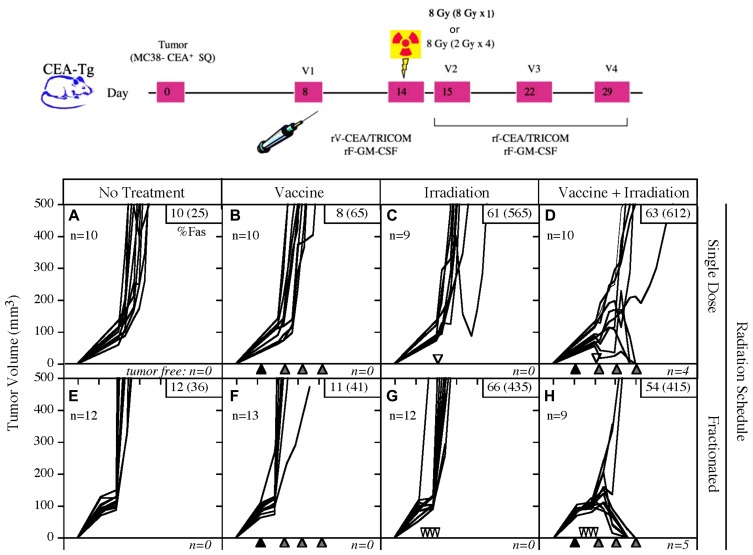
**Combination of single-dose or fractionated RT with vaccine therapy.** Mice transgenic for CEA were implanted subcutaneously on day 0 with the MC38-CEA tumor cell line, then randomized to receive either no treatment, vaccine alone, EBRT alone, or the combination of vaccine and EBRT. The vaccine consisted of poxviral vectors expressing CEA and TRICOM (rF/V-CEA/TRICOM). All vaccines were coadministered with a poxviral vector expressing GM-CSF. RT was administered either as a single dose (8 Gy on day 14) or fractionated (2 Gy on days 11, 12, 13, and 14). Neither modality was effective alone, but the combination of vaccine with single-dose or fractionated RT was curative in 40 and 55% of mice, respectively. (Adapted from Chakraborty et al., 2004.)

A number of preclinical studies have revealed that RT acts synergistically with active therapeutic vaccination to enhance antitumor immune responses. Chakraborty et al. (2004) focused on the combination of 8 Gy EBRT delivered directly to the tumor in combination with a vaccine composed of vaccinia and fowlpox vectors that express CEA and a triad of costimulatory molecules: B7-1, ICAM-1, and LFA-3 (rV/F-CEA/TRICOM). Although either treatment alone was ineffective at reducing tumor burden, the combination of EBRT and vaccine was not only curative in 50% of mice bearing CEA-expressing tumors, but also imparted protection from subsequent tumor challenge (**Figure 2**; Chakraborty et al., 2004). Notably, mice cured of tumors demonstrated antigen cascade, developing CD4 and CD8 T cell responses not only to CEA, but also to other tumor antigens not encoded in the vaccine, such as gp70 (Chakraborty et al., 2004). They reported that the immune response to gp70 was markedly greater than that seen to the antigen encoded in the vaccine, suggesting that the immune response to the cascade antigens may play an important role in the observed antitumor activity. Results from this preclinical study provided the rationale to evaluate the use of EBRT and therapeutic cancer vaccines in the clinic.

**FIGURE 3:**
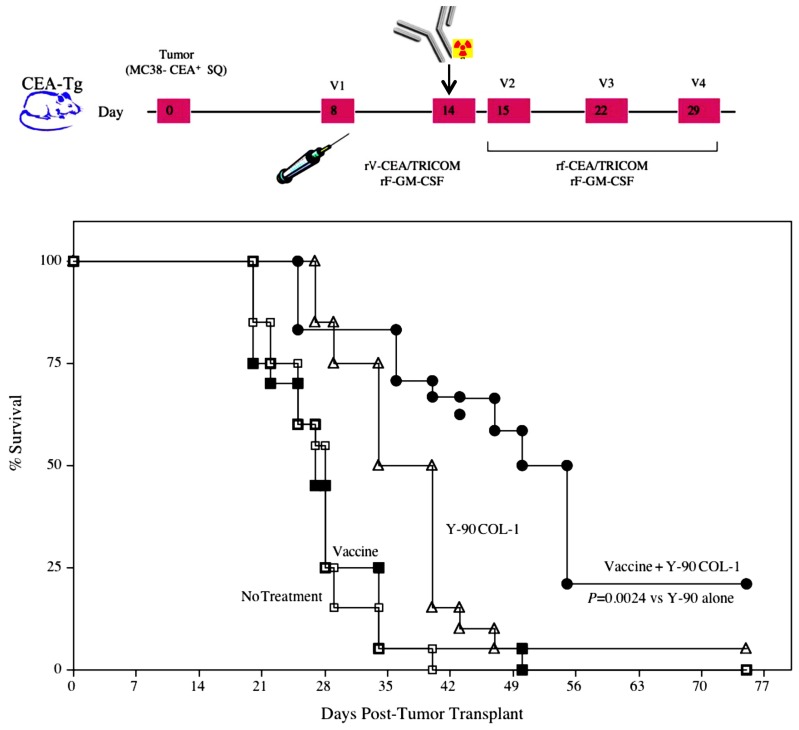
**Combination of a radiolabeled mAb with vaccine increased survival in tumor-bearing mice.** Mice transgenic for CEA were implanted subcutaneously on day 0 with the MC38-CEA tumor cell line. A control group (open squares) received HBSS buffer only. A second group (closed squares) received a vaccine consisting of poxviral vectors expressing CEA and TRICOM (rV/F-CEA/TRICOM). All vaccines were coadministered with a poxviral vector expressing GM-CSF. A third group (open triangles) received RT consisting of 100 μCi yttrium-90-labeled anti-CEA mAb (Y-90-labeled COL-1) intravenously on day 14. A fourth group (closed circles) received the combination of vaccine plus radiolabeled mAb. Mice were monitored weekly for tumor size and survival. (Adapted from Chakraborty et al., 2008a.)

Therapeutic synergy has also been reported utilizing vaccine-mediated immunotherapy combined with radiolabeled mAb. mAbs can guide radionuclides to cancer cells, precisely and preferentially target tumor cells, and seek out micrometastases that are unobservable by current imaging technology and cannot be targeted by EBRT. A recent study cited the ability of radiolabeled mAb to alter tumor-cell phenotype and enhance immunologic targeting of tumor cells (Chakraborty et al., 2008a). In that study, mice transgenic for CEA were transplanted with MC38-CEA tumor cells, then treated with yttrium-90-labeled anti-CEA mAb alone or in combination with CEA-targeted vaccine therapy. A single dose of yttrium-90-labeled anti-CEA mAb, in combination with vaccine, statistically increased survival in tumor-bearing mice relative to vaccine or mAb therapy alone (**Figure 3**; Chakraborty et al., 2008a). Of note, mice receiving the combination therapy also had a marked increase in the percentage of viable tumor-infiltrating CEA-specific CD8 T cells relative to vaccine alone, demonstrating that these cells were unaffected by the residential radiation source. Similar to what was noted with EBRT, mice cured of tumors demonstrated an antigen cascade, resulting in CD4 and CD8 T cell responses not only for CEA, but also for tumor antigens not encoded in the vaccine.

Brachytherapy, yet another form of clinically relevant RT, has also been evaluated in combination with vaccine-mediated immunotherapy. Brachytherapy, which involves implanting a radiation source such as iodine-125 into or near the site of a malignant tumor to target tumor cells with continuous high-dose radiation, has also been shown to alter the phenotype of tumor cells. A single study demonstrated the ability of iodine-125 to increase the expression of Fas >2-fold in tumors relative to sham-treated mice (Hodge et al., 2012). In this study, CEA-transgenic mice were implanted with a Lewis lung carcinoma cell line expressing CEA (LL2-CEA) both subcutaneously and intravenously. Mice received either no treatment, brachytherapy alone in which iodine-125 seeds were implanted near the subcutaneous tumor, vaccine alone, in this case a diversified prime and boost of poxviral vectors expressing gp70 and TRICOM, or the combination of brachytherapy and vaccine. The only therapeutic regimen that suppressed the number of pulmonary metastases in this model was the combination of brachytherapy (directed at the primary subcutaneous tumor alone) with vaccination (Hodge et al., 2012). Thus, the abscopal effect only occurred in mice treated with the combination of brachytherapy and vaccine. A recent study by Dewan et al. (2009) similarly noted that RT induced an abscopal effect only when used in combination with immunotherapy. In their study, they noted that fractionated local radiotherapy to one palpable tumor synergized with CTLA-4 blockade to induce antitumor T cell immunity and inhibit the growth of a second palpable tumor outside the radiation field.

## CLINICAL EVIDENCE OF THE EFFICACY OF COMBINED RADIATION AND IMMUNOTHERAPY

Results from the preclinical studies described above and from additional reports as well have provided the rationale for clinical evaluation of the combination of RT and cancer immunotherapy. In a phase I study of patients with advanced hepatoma, participants were given 8 Gy of radiation, followed 2 days later by an intratumoral injection of autologous immature DCs. Of 10 patients evaluated for immune response, six showed increased natural killer cell activity, eight had increases in alpha-fetoprotein (AFP)-specific immune responses by cytokine-release assay, and seven showed increased AFP-specific immune responses by ELISPOT. Of the 14 patients who entered the trial, four had minor responses and two had partial responses, including a patient who had a decrease in AFP from 128 to 1.6 ng/mL (Chi et al., 2005).

A randomized phase II study in men with localized prostate cancer evaluated the use of a recombinant poxviral-based vaccine expressing PSA combined with standard definitive radiotherapy (Gulley et al., 2005). Patients in the combination arm received a priming vaccine of recombinant vaccinia (rV) expressing PSA (rV-PSA) admixed with rV expressing the costimulatory molecule B7-1. This was followed by monthly boosts with recombinant fowlpox (rF)-PSA. The vaccines were administered with local granulocyte–macrophage colony-stimulating factor and low-dose systemic IL-2 (4 million IU/M^2^). Two courses of EBRT were given daily for 5 days, with a 2-day holiday between the fourth and sixth vaccinations. Results from this clinical trial indicated that the combination was safe, well tolerated, and, more importantly, effective at generating PSA-specific immune responses. Approximately 76.5% of patients (13 of 17) in the combination therapy arm showed a ≥-fold increase in PSA-specific T cells vs. 0% (0 of 8) in the radiation-alone arm (*P* < 0.0005). In addition, six of eight patients developed post-treatment T cell responses specific for at least one additional endogenous TAA not encoded by the vaccine, indicating the presence of antigen cascade. These included the generation of T cells against PSMA, PAP, prostate stem cell antigen (PSCA), and/or MUC-1 (**Table 1**). In some cases the immune response to a cascade antigen was even greater than the response to PSA. There were no significant changes in the patients’ responses to flu peptide, and all patients remained negative for responses to HIV. Only grade 2 toxicities were related to the vaccine itself; however, some grade 3 toxicities were attributed to IL-2. A follow-up study was conducted to evaluate the use of a metronomic dose of IL-2 (0.6 million IU/M^2^) in order to reduce some of the toxicity seen in the previous trial (Lechleider et al., 2008). This study used the same vaccination schedule as the previous trial, except that RT was administered following the third booster vaccination instead of the fourth. Patients in this trial experienced less toxicity attributable to IL-2 and developed similar immune responses (**Table 2**). A third trial was conducted evaluating the combination of the rV/F-CEA/TRICOM vaccine with EBRT delivered directly to liver metastases in patients with CEA^+^ solid tumors (Gulley et al., 2011). Twelve patients, 11 with CEA^+^ colon cancer and 1 with CEA^+^ rectal cancer, received a priming vaccination with rV-CEA/TRICOM on day 1, with biweekly booster vaccinations with rF-CEA/TRICOM. Four 8-Gy courses of EBRT were delivered to sites of liver metastasis 1 day following booster vaccinations. Unfortunately, the design of this study was not optimal for assessing the ability of radiation to enhance the clinical benefit of vaccine treatment strategies. Of the two evaluable patients, neither showed an increase in CEA-specific T cells above baseline after therapy.

**Table 1:** Immune responses following treatment with poxviral vaccines expressing PSA and B7-1 in combination with EBRT and low-dose IL-2 (Gulley etal., 2005).

Patient	Sample	PSA	PSMA	PAP	PSCA	MUC-1
3	pre	ND	ND	ND	ND	ND
	post 3	1/50,000	ND	1/85,714	1/85,714	1/23,077
	post 8	1/46,154				
6	pre	ND	ND	ND	ND	ND
	post 3	1/54,545	1/85,714	ND	ND	1/60,000
	post 8	1/22,222				
7	pre	ND	ND	ND	ND	ND
	post 3	1/42,857	1/200,000	1/85,714	ND	ND
	post 8	1/15,000				
8	pre	ND	ND	ND	–	1/80,000
	post 3	ND	1/62,500	ND	–	1/46,154
	post 8	1/66,667				
11	pre	1/100,000	ND	ND	ND	ND
	post 3	1/85,714	ND	ND	ND	1/40,000
	post 8	ND				
12	pre	1/100,000	ND	1/200,000	1/200,000	ND
	post 3	1/150,000	ND	ND	ND	1/35,294
	post 8	1/200,000				

*ND, none detected (<1/200,000). Samples obtained after indicated vaccine cycle.*

**Table 2:** Immune responses following vaccination with poxviral vaccines expressing PSA and B7-1 in combination with EBRT and metronomic IL-2 (Lechleider etal., 2008).

Patient	Sample	PSA	MUC-1	PAGE-4	XAGE-1
31	pre	ND	1/85,714	ND	ND
	post 3	ND	ND	ND	ND
	post 3 + 2	1/45,455	–	–	–
	post 5 + 2	–	ND	ND	ND
	post 8	1/60,000	1/37,500	ND	1/27,273
32	pre	1/120,000	ND	1/100,000	1/23,077
	post 3	1/17,391	ND	1/80,000	1/28,571
	post 3 + 2	ND	–	–	–
	post 5 + 2	–	ND	1/22,222	1/46,154
	post 8	ND	ND	1/100,000	1/50,000
33	pre	–	ND	ND	ND
	post 3	–	ND	1/200,000	1/54,545
	post 5	–	ND	ND	ND
	post 8	–	1/46,154	ND	1/24,000
34	pre	ND	–	–	–
	post 3	1/46,154	–	–	–
	post 5 + 3	ND	–	–	–
	post 8	ND	–	–	–
37	pre	1/150,000	–	–	–
	post 2	ND	–	–	–
	post 5	1/12,000	–	–	–
38	pre	ND	–	–	–
	post 3	1/85,714	–	–	–
	post 8	1/28,462	–	–	–

*PAGE-4 and XAGE-1 denote members of the PAGE/GAGE family of prostate cancer TAAs. ND, none detected (<1/200,000). Samples obtained after indicated vaccine cycle (i.e., post 3 + 2 = 2 months after cycle 3).*

The combination of ^153^Sm-EDTMP and vaccine is also currently being studied in a randomized phase II trial in patients with castration-resistant prostate cancer (CRPC) metastatic to bone (Heery et al., 2012). The primary endpoint of the trial is to determine if ^153^Sm-EDTMP combined with vaccine can improve time to progression over ^153^Sm-EDTMP alone. Patients will receive 1 mCi/kg ^153^Sm-EDTMP alone or in combination with an rV/F-PSA/TRICOM vaccine (PROSTVAC^®^, Bavarian Nordic) administered in a biweekly diversified prime/boost regimen for the first three vaccinations starting on day 1, then monthly thereafter. ^153^Sm-EDTMP will be administered on day 8, then every 12 weeks thereafter. Currently, 37 of a projected 68 patients have been enrolled. Interim analysis determined that at 4 months, 5 of 17 patients (29.4%) receiving combination therapy remained progression-free, while only 2 of 17 (11.8%) remained progression-free on ^153^Sm-EDTMP alone. The median time to progression was 60 days in the ^153^Sm-EDTMP-alone group and 117 days in the combination group. This early indication of improved time to progression supports the continuation of this trial, allowing for the evaluation of secondary endpoints of immunogenic stimulation and overall survival.

In addition to vaccines, RT has also been evaluated clinically in combination with additional types of immunotherapy. Three trials have been undertaken to determine if RT can enhance the antitumor efficacy of IPI in patients with metastatic CRPC. In all three trials, single-fraction RT was given just prior to the start of IPI therapy which was given at doses of either 3 or 10 mg/kg once every 3 weeks for four cycles. All three trials determined that the combination was well tolerated, but similar reductions in PSA were observed in the IPI treatment groups regardless of the addition of RT (Beer et al., 2008; Slovin et al., 2009, 2012). Additional trials examining the timing of RT with respect to IPI treatment may lead to a combination treatment that acts synergistically similar to that reported in metastatic melanoma (Postow et al., 2012). A recent single arm phase I/II trial examined the efficacy of combining low-dose RT (4 Gy over 2 days) with administration of the TLR9 agonist PF-3512676 to 15 patients with low-grade B cell lymphoma (Brody et al., 2010). In this trial, PF-3512676 was administered via intratumoral injection to the same site as local RT. PF-3512676 was administered immediately prior to the first dose of radiation, immediately following the second and then weekly for 8 weeks. The combination was well tolerated and resulted in one complete response, three partial responses, and two patients having stable/regressing disease. Responding patients displayed increases in tumor-reactive CD8^+^ T cells and a reduction in Tregs. Another recent phase I trial examined the combination of stereotactic body RT with systemic IL-2 therapy for the treatment of metastatic melanoma and renal cell carcinoma (RCC; Seung et al., 2012). Twelve patients (seven with melanoma, five with RCC) received one, two, or three doses of 20 Gy stereotactic body RT with bolus IL-2 (600,000 IU/kg) beginning 3 days following the final dose of RT. IL-2 was given every 8 h for a maximum of 14 doses with a second cycle of treatment occurring 2 weeks later. By positron emission tomography, five patients with melanoma and one with RCC achieved a complete response while two additional RCC patients achieved a partial response. Responding patients exhibited a higher frequency of early-activated effector memory CD4^+^ T cells in the peripheral blood. Both of these studies support the immunomodulatory activity of RT and its combination with additional forms of immunotherapy. Additional trials, however, still need to be performed to determine the extent of the increased efficacy of these combinations.

## PERSPECTIVES ON THE FUTURE OF COMBINED RADIATION AND IMMUNOTHERAPY FOR THE TREATMENT OF CANCER

The goal of cancer immunotherapy is to overcome tolerance to weakly immunogenic TAAs and to stimulate an immune response to tumor cells. Ionizing radiation induces tumor-cell death, thereby releasing the multiple novel tumor antigens required to overcome tolerance and igniting the “danger signals” needed to stimulate an immune response. RT may be able to overcome the ability of cancer cells to escape immune recognition and therefore act synergistically with immunotherapy to enhance immune responses, inhibit immunosuppression, and/or alter the phenotype of tumor cells, rendering them more susceptible to immune-mediated killing. Preclinical studies have shown that RT from a variety of different sources cannot only induce tumor-cell death in a manner consistent with antitumor immune activation, but can also phenotypically modify tumor cells not killed by RT in a way that facilitates both immune recognition and immune-mediated killing. Capitalizing on the immunologic effects induced by RT by adding potent antitumor immunotherapy agents may lead to synergistic approaches to cancer management that offer feasible, well-tolerated therapeutic options for cancer patients.

Questions remain, however, as to how best to exploit the largely untapped resource of radiation and immunostimulatory combination therapy. First, although many modes of RT have been shown to induce similar alterations in tumor phenotype and microenvironment, there may be subtle variations in the induction of these responses brought about by a given type of RT. These variations may be better exploited by a specific type of cancer immunotherapy, including those discussed herein or other emerging immunotherapies such as the vaccine sipuleucel-T (Provenge^®^, Dendreon Corp.). Second, as discussed here, combining a specific dosage and course of RT with immunotherapy may be more efficacious at enhancing clinical benefit; this concept, however, needs further investigation. Along these same lines, the timing of administration of RT and immunotherapy during combination treatment also needs further investigation. As discussed, administering immunotherapy prior to RT allows for the generation of a memory immune response that is less susceptible to immunodepletion brought about by RT. Although it was not designed for definitive determination, one may infer from the trial evaluating rV/F-CEA/TRICOM with EBRT delivered directly to liver metastases in patients with CEA^+^ solid tumors that 1 day post-vaccination is too soon for RT. On the other hand, administering immunotherapy following RT may take advantage of the homeostatic peripheral expansion of the immune compartment that occurs following some types of RT. As with phenotypic and microenvironmental changes, the timing of each therapy may depend on the specific therapies being combined. The final consideration concerning the combination of RT and immunotherapy for the treatment of cancer is identifying the most appropriate patient population. EBRT and vaccine combination trials in prostate cancer may have yielded more positive results because definitive RT for localized prostate cancer does not involve extensive lymph node irradiation, thus sparing much of the patients’ lymphocyte population. This could suggest that combination therapy should be examined further in the setting of RT that avoids extensive lymph node irradiation. However, in the prostate cancer trials discussed here, some patients developed more stable immune responses that were less susceptible to blunting by RT than others. The reason for this result is unclear, however, indicating that further studies are required to determine which patient population would benefit most from this combination therapy. In addition, it will also be important to determine the stage of disease at which this combination will be most beneficial to the patient. As monotherapies, both immunotherapy and radiation may be insufficient to eliminate bulky tumor masses or an entire metastatic burden. Even though combination therapy may be more effective in this advanced state, patients with smaller primary tumors and lower metastatic burdens may derive greater clinical benefit due to the lower tumor burden needed to be overcome. Additional clinical trials in earlier disease settings will be needed to confirm this approach.

Substantial preclinical evidence has revealed a synergistic relationship between RT and immunotherapy. Anecdotal evidence and prospective clinical data also support the efficacy of this treatment regimen. As most of the studies reviewed here have focused on an immunological response as the primary endpoint, further clinical trials are needed to determine if adding active immunotherapy to definitive RT can affect clinical outcomes. Learning how best to exploit radiation-induced immunogenic changes in cancer patients with the addition of active immunotherapy is an exciting frontier in cancer therapy research, and has the potential to greatly improve patient care in the future.

## Conflict of Interest Statement

The authors declare that the research was conducted in the absence of any commercial or financial relationships that could be construed as a potential conflict of interest.
